# Construction of an associative model for prolonged intensive care unit stay in sepsis patients combined with myocardial injury

**DOI:** 10.1016/j.clinsp.2026.101074

**Published:** 2026-08-13

**Authors:** Dabang Lei, Xianfei Ke, Minglang Liao, Kai Yang

**Affiliations:** Department of Emergency and Critical Care Center, Renmin Hospital, Hubei University of Medicine, Shiyan, Hubei, PR China

**Keywords:** Length of stay, Sepsis, Myocardial injury, Machine learning

## Abstract

**Objective:**

This study aimed to identify the factors contributing to Prolonged Length of Stay (PLOS) in intensive care units for sepsis patients combined with Myocardial Injury (MI) and to construct an associative model.

**Methods:**

Data were from the Medical Information Mart for Intensive Care IV database. Variables were selected using the Least Absolute Shrinkage and Selection Operator (LASSO) regression analysis. The performance of five Machine Learning (ML) models established based on key factors, including the Logistic model, XGBoost, LightGBM, AdaBoost, and RandomForest, was compared by 10-fold nested cross-validation. The optimal associative model performance was validated by 10-fold cross-validation repeated 5-times.

**Results:**

Among 1792 sepsis patients combined with MI, 448 patients developed PLOS. LASSO regression analysis indicated that the Sequential Organ Failure Assessment score, potassium, age, heart rate, systolic blood pressure, red blood cell, acute kidney injury, vasopressor, mechanical ventilation, and continuous renal replacement therapy might be factors related to PLOS. Combining the results of 10-fold nested cross-validation, the Logistic model, which included the 10 variables, was more stable than the other four ML models. The mean Area Under the Curves (AUCs) for the training and validation sets by 10-fold cross-validation repeated 5-times were 0.852 (0.849‒0.856) and 0.848 (0.837‒0.857). The AUC of the test set was 0.846 (0.796‒0.890).

**Conclusion:**

PLOS in sepsis patients combined with MI involved multiple influences. Early identification of high-risk factors and intensive multidisciplinary treatment can help to shorten LOS and reduce the risk of complications.

## Highlights

• Identifies key associative factors for prolonged ICU stay in sepsis patients with MI.
• Logistic regression demonstrated stable performance compared to four ML models.
• Potassium and RBC levels at admission influenced the baseline predictive model.
• Using the associative (with treatments) and early predictive (baseline-only) models.
• Repeated cross-validation supported model robustness in internal validation.

## Introduction

Sepsis is a heterogeneous clinical syndrome caused by microbial infection. The syndrome can lead to severe systemic infections with complications such as hypotension, multi-organ failure, shock, and even death.^1^ Recent estimates suggest that sepsis affects nearly 50 million people globally each year, and its high mortality rate and high healthcare expenditures pose a serious challenge to public health systems.^2^ The prevalence of sepsis patients combined with Myocardial Injury (MI) is approximately 40%‒50%.^3^ Sepsis patients combined with MI not only increase the complexity of the disease but are also strongly associated with short- and long-term mortality.^4^ Furthermore, in the United States, direct and indirect medical expenditures exceed 38 billion dollars due to sepsis, underscoring its significant impact on healthcare resource utilization.^5^ This burden is further exacerbated by Prolonged Length of Stay (PLOS), which is closely linked to excessive use of hospital resources and escalating healthcare expenses. Moreover, PLOS heightens the risk of in-hospital complications for patients, potentially worsening their prognosis, increasing mortality rates, and diminishing their care experience.^6^^,^^7^ Therefore, early assessment of PLOS risk in sepsis patients with MI is crucial for implementing timely preventive measures and improving clinical outcomes.

Machine Learning (ML) is a core technology of Artificial Intelligence. It has shown great potential in the medical field. ML is based on an algorithmic framework that continuously optimizes discriminative performance through feature extraction and adaptive learning of data patterns.^8^ Its applications have permeated various fields, including disease risk prediction^9^ and medical big data mining.^10^ In clinical practice, ML models offer significant value by enabling early detection, risk stratification, and mortality prediction, thereby supporting personalized decision-making.^11^ In sepsis management, ML is currently employed for these purposes. Although research has been conducted to explore the influencing factors of LOS using ML models,^12^ studies discriminating PLOS in sepsis patients combined with MI remained very limited. Therefore, there is an urgent need to construct an associative model based on ML algorithms to discriminate the PLOS of sepsis patients combined with MI. Exploring the factors associated with the PLOS of sepsis patients combined with MI patients and their associated models may effectively reduce their hospital stay duration and healthcare costs.

## Methods

### Study design and study setting

The retrospective study followed the STROBE statement and was established based on sepsis patients combined with MI admitted to the ICU for the first time in the Medical Information Mart for Intensive Care IV (MIMIC-IV) data. One researcher in this study obtained permission from MIMIC-IV (certification number: 55,710,882). The MIMIC-IV database collects clinical data on >190,000 patients admitted to the Beth Israel Deaconess Medical Center from 2008 to 2019, with 450,000 hospitalizations recorded. The database has been rigorously de-identified with all patient information before public release to ensure that patient privacy is fully protected. Therefore, there is no need to obtain ethical review board approval and informed patient consent.

### The definition of sepsis patients combined with MI

According to the Third International Consensus Definition of Sepsis and Infectious Shock (Sepsis-3), the diagnostic criteria for sepsis are the presence of a suspected infection in the patient and an increase of ≥ 2-points in the Sequential Organ Failure Assessment (SOFA) score from the baseline level.^13^ Studies indicate that cardiac Troponin T (cTnT) levels are strongly associated with the prognosis of MI, and elevated cTnT can reflect the presence of MI in patients with sepsis.^14^ In the MIMIC-IV database, the 99th percentile reference upper limit for cTnT is 0.01 ng/mL.^3^ This study adopted cTnT levels ≥0.01 ng/mL as a criterion for the determination of MI and used it as a diagnostic threshold for sepsis patients combined with MI.

### Inclusion and exclusion criteria

**Fig. 1:**
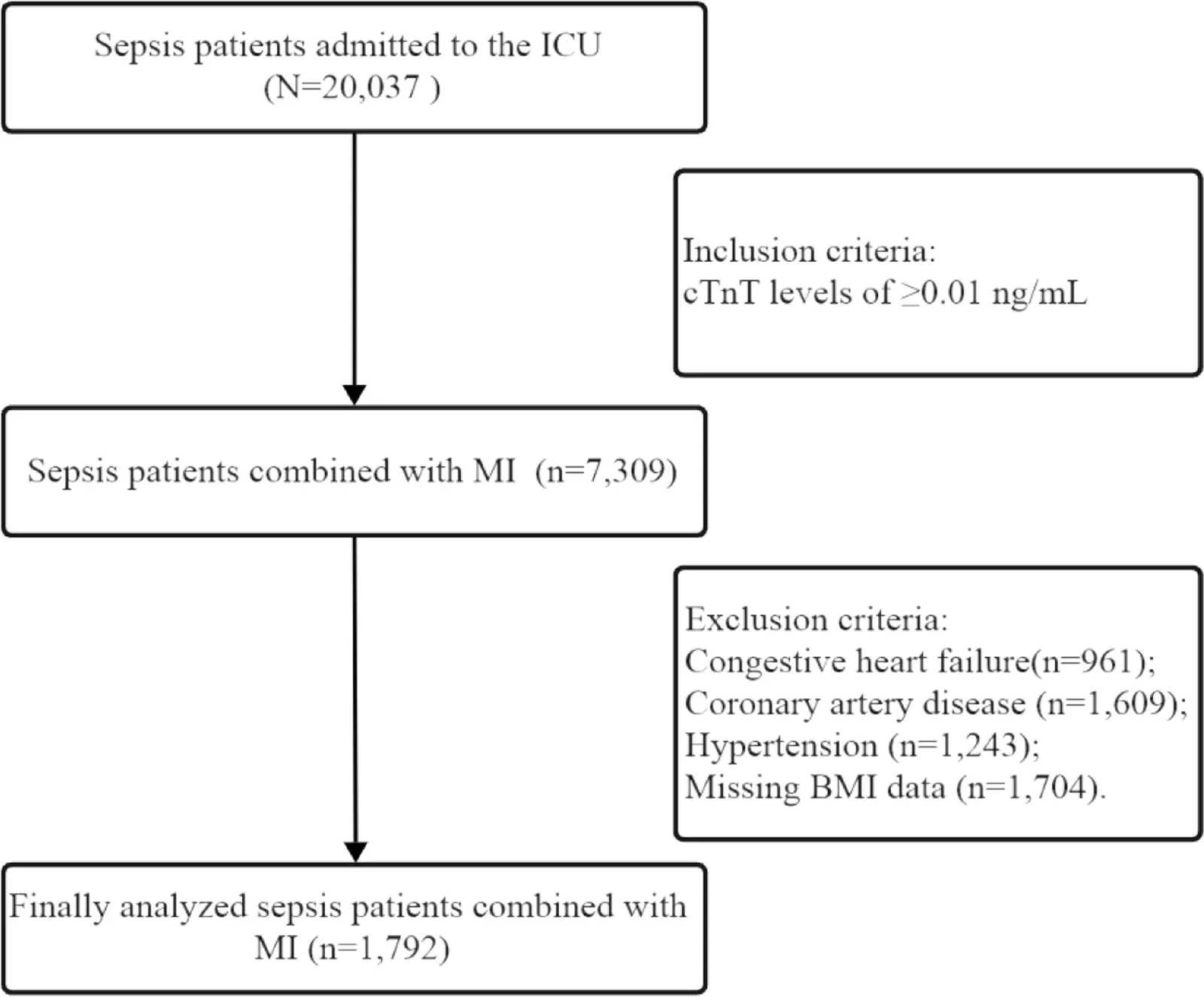
The flowchart of participants.

One of the authors of this study received permission to access and extract relevant data from the MIMIC-IV database. Researchers can screen the clinical information of patients of interest based on certain exclusion criteria. Initially, 20,037 septic patients admitted to the ICU were retrieved from the MIMIC-IV database, with 7309 having cTnT levels of ≥ 0.01 ng/mL. Patients with congestive heart failure (n = 961), coronary artery disease (n = 1609), and hypertension (n = 1243) that may cause elevated cTnT were excluded. Those with missing BMI data (n = 1704) were also excluded. Eventually, 1792 sepsis patients combined with MI were included in this study. Fig. 1 illustrates the inclusion process of the study population.

### Study outcomes

The outcome of this study was the PLOS in the ICU for sepsis patients combined with MI. There is no consensus among different studies on the choice of PLOS cut-off point, which is commonly based on 14-days or the 75th percentile.^15^ To better align with the ICU stays of the study subjects, the length of ICU stay was categorized into non-PLOS and PLOS groups based on the 75th percentile (10.187 days).

### Collected variables

Patient demographic characteristics were extracted from the database as follows: age (years), gender, and Body Mass Index (BMI, kg/m^2^). Patient health condition variables included Acute Kidney Injury (AKI), Chronic Obstructive Pulmonary Disease (COPD), diabetes, septic shock, SOFA score, and Simplified Acute Physiology Score II (SAPSII). Treatments included vasopressor, aspirin use, Mechanical Ventilation (MV), and Continuous Renal Replacement Therapy (CRRT). Common signs and laboratory tests included Heart Rate (HR, beats/min), Systolic Blood Pressure (SBP, mmHg), Diastolic Blood Pressure (DBP, mmHg), percutaneous arterial oxygen saturation (SpO_2_, %), Respiratory Rate (RR, breaths/min), glucose (mg/dL), potassium (mEq/L), pondus Hydrogenii (pH), partial pressure of arterial oxygen (PaO_2_, mmHg), partial pressure of carbon dioxide (PCO_2_, mmHg), platelets (K/μL), White Blood Cell (WBC, K/μL), anion gap (AG, mEq/L), chloride (mEq/L), creatinine (mg/dL), International Normalized Ratio (INR), Prothrombin Time (PT, second), Red Blood Cell Count (RBC, m/μL) and Blood Urea Nitrogen (BUN, mg/dL). Laboratory variables were assessed upon initial admission to the ICU, while treatment data were obtained during the ICU. The authors additionally incorporated diuretics and estimated Glomerular Filtration Rate (eGFR) into sensitivity analyses examining the impact on potassium levels.

### Statistical analysis

The Shapiro-Wilk test was used to assess data normality in this study. Data following a normal distribution were described using mean ± standard deviation, with group comparisons made using the *t*-test. Data not conforming to a normal distribution were described using medians and quartiles (P25‒P75), with group comparisons made using the two-group rank sum test. Categorical variables were described using frequencies and percentages, with group comparisons made using the Chi-Square test or Fisher’s exact probability method. Due to missing data in some cases (Table S1), continuous variables with < 25% missing data were analyzed using the multiply interpolated ‘mice’ package. Least Absolute Shrinkage and Selection Operator (LASSO) regression analysis was used to perform variable selection on the imputed data via “glmnet” package. Collinearity diagnostics via “rms” package were utilized to determine collinearity between the variables, with collinearity being acknowledged when the Variance Inflation Factor (VIF) > 10. Two multivariable logistic regression analyses were applied to explore the influencing factors of PLOS. Model 1 included baseline information, and Model 2 included baseline information and treatments. The nomogram models were constructed to visualize the results via “rms” package. In this study, the PLOS grouping was based on the 75th LOS as the cutoff point. The authors validated the model stability using a 14-day LOS cutoff point. Additionally, excluding patients with hypertension, CAD, or CHF artificially purified the cohort but may have compromised clinical applicability. Therefore, the authors included this group of patients in dataset 2 to validate the model’s applicability.

All patients were randomly assigned to either the training set or the test set based on an 8:2 ratio. To mitigate overfitting and obtain a robust estimate of performance, 10-fold nested cross-validation was conducted on the training set. The inner loop was used for hyperparameter tuning via grid search, while the outer loop provided an unbiased performance estimate. Based on the “glmnet” package, “xgboost” package, “lightgbm” package, “adabag” package, and “randomForest” package, various models, including Logistic model, XGBoost, LightGBM, AdaBoost, and RandomForest, were trained. The average performance of each model in terms of Area Under the Curve (AUCs), accuracy, sensitivity, specificity, Positive Predictive Value (PPV), Negative Predictive Value (NPV), and F1 score was calculated. Based on “pROC” package and “rmda” package, the Receiver Operating Characteristic (ROC) curves and Decision Curve Analysis (DCA) were used to determine the discriminative performance of the associative models and the net clinical benefit. Ten-fold cross-validation repeated 5-times was employed to validate the robustness of the optimal model. Subsequently, the model containing only baseline information was employed as a predictive model. The predictive model was interpreted using the Shapley Additive explanations (SHAP) algorithm via “fastshap” package, which assigns a SHAP value to each variable to quantify its impact on predictive accuracy. Based on “ggbeeswarm” package, a SHAP summary plot was created to illustrate the contribution of each feature to model performance. Restricted Cubic Spline (RCS) analysis with three nodes was employed to investigate the non-linear relationship between variables and PLOS via “rms” package. All of the above statistical analyses were processed using R software (https://posit.co/download/rstudio-desktop/); p < 0.05 was considered statistically significant.

### The introduction of the ML model

The logistic model is a traditional single-model classification algorithm mainly used to explore the relationship between features and the probability of binary classification results, which is widely cited in the medical field due to its ability to compute the odds ratio.^16^ In contrast, RandomForest, XGBoost, LightGBM, and AdaBoost are integrated models based on decision trees, where a more powerful model is constructed by combining multiple single models, thus integrating the advantages of each model to achieve better performance. The RandomForest algorithm embodies the idea of group intelligence by creating different training sets through random row sampling and column sampling and growing the tree using the bootstrap method to reduce training variance and improve the generalization of the model. The algorithm has high discriminative accuracy and variable importance information for classification and can be used without tuning parameters.^17^ XGBoost, LightGBM, and AdaBoost are based on the idea of gradient boosting and achieve stronger learning by integrating multiple weak learners. XGBoost is highly flexible and scalable by performing a second-order Taylor expansion of the loss function to prevent overfitting and using the structural score of the tree to determine the splitting point, as well as using parallel and distributed computation to improve efficiency.^18^ LightGBM uses a histogram approximation algorithm to discretize the continuous features and split by boosting the largest leaf node, and uses the GOSS technique to prioritize samples with larger gradients, thus making the optimal splitting point faster and improving the training efficiency.^19^ AdaBoost is a nonlinear ML algorithm that constructs a tree by adjusting the weights of the samples and combines multiple trees to form a strong classifier, which does not require feature screening, can automatically select features, and has a low risk of overfitting^20^

## Results

### Participant basic information

The present study evaluated the characteristics of 1792 sepsis patients combined with MI, with a median age of 59-years, comprising 55.636% male patients and 44.364% female patients. There were 448 patients in the PLOS group and 1344 patients in the non-PLOS group. A comparison of the basic information of the two groups showed that there were differences in some demographic characteristics, health-related factors, and biochemical indices (Tables S2 and S3). Patients in the PLOS group were younger than those in the non-PLOS group (57 vs. 60). Regarding health-related conditions and treatments, the PLOS group had a higher incidence of septic shock (37.054%vs. 20.759%) or AKI (95.205%vs. 69.568%), and more patients received vasopressor (68.080%vs. 36.086%), MV (39.732%vs. 9.301%), or CRRT (24.777%vs. 4.613%) treatments. Health measures indicated higher SOFA (4.000 vs. 3.000), SAPS II (42.000 vs. 38.000), and HR (78.865 vs. 74.779) but lower SBP (84.000 vs. 86.000) in the PLOS group. Biochemical differences included lower potassium (3.100 vs. 3.400), pH (7.280 vs. 7.320), RBC (2.310 vs. 2.840), and BUN (11.000 vs. 13.000) in the PLOS group. In addition, WBC (10.500 vs. 9.100) was higher in the PLOS group than in the non-PLOS group (all p < 0.05).

### The influencing factors of PLOS in sepsis patients combined with MI

**Fig. 2:**
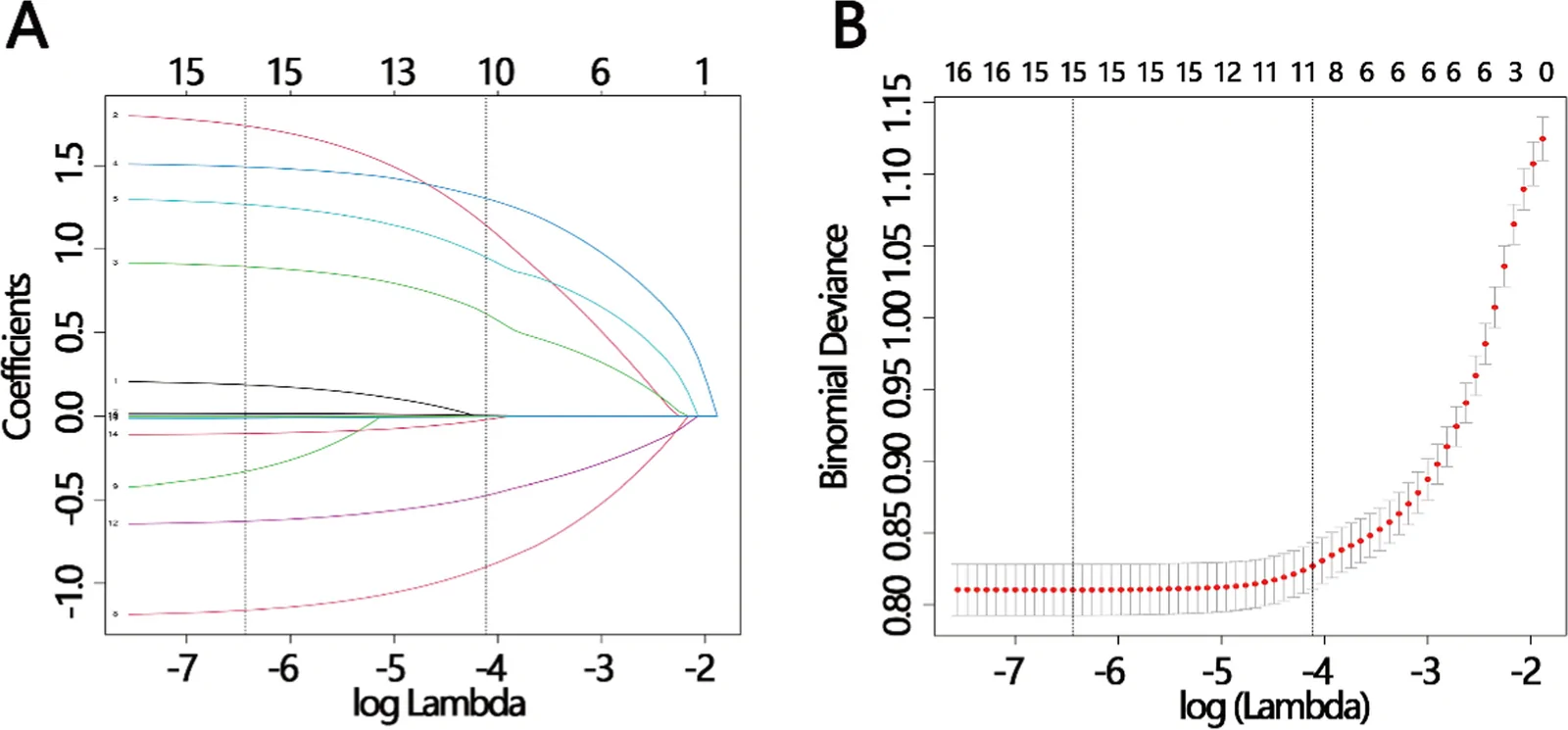
LASSO regression analysis of 16 variables. (A) Variable coefficients in the LASSO analysis. (B) Cross-validation plot with the optimal λ determined by the 1-SE rule (right dashed line). The SE in this analysis was 0.016. LASSO, Least Absolute Shrinkage and Selection Operator; SE, Standard Error.

**Fig. 3:**
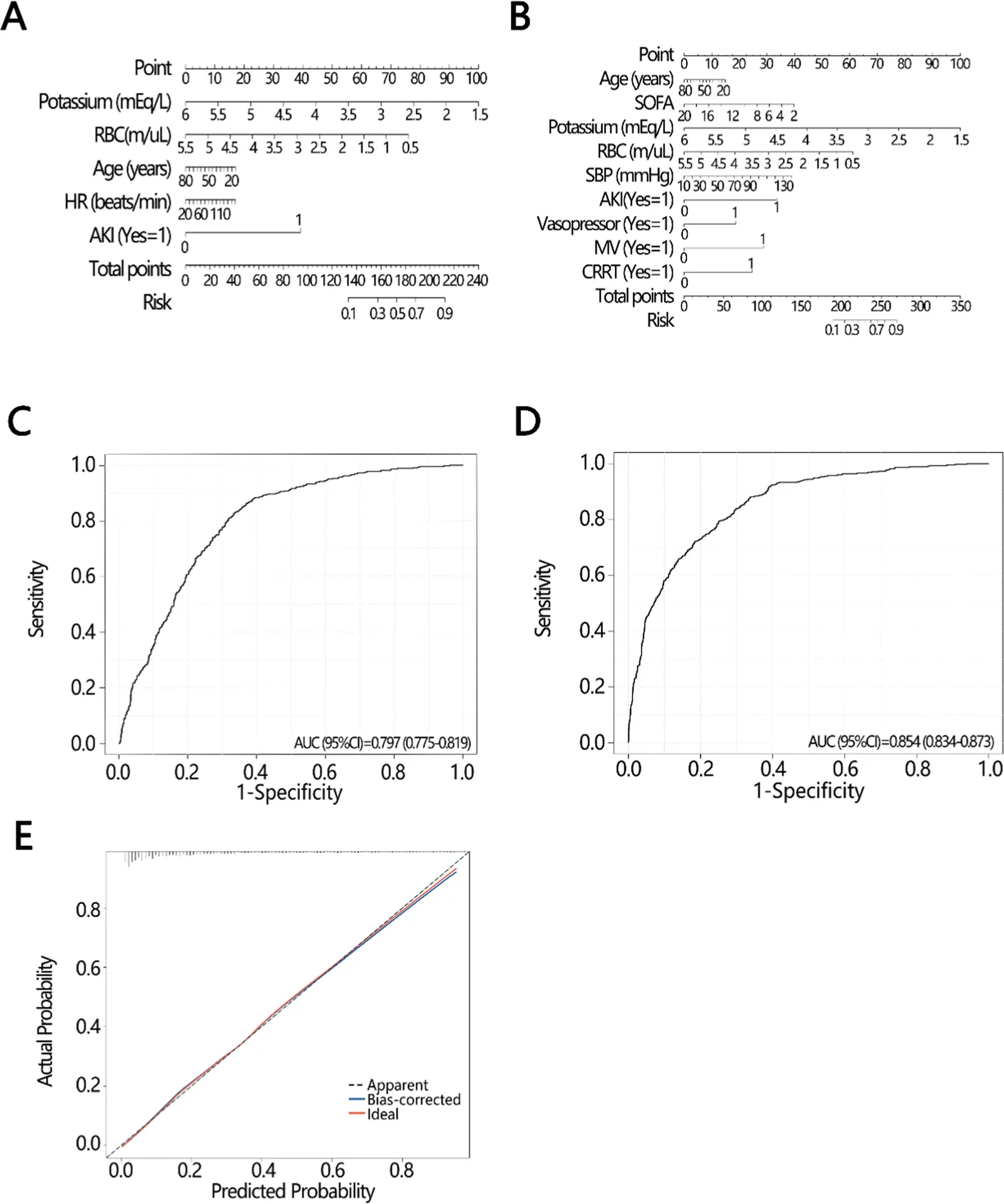
Construction and evaluation of nomogram models based on the results of multivariable logistic regression. (A) Visualization of the PLOS nomogram 1 model; (B) Visualization of the PLOS nomogram 2 model; (C) The ROC curve of the nomogram 1 discriminative model; (D) The ROC curve of the nomogram 2 discriminative model; (E) The calibration curve of the nomogram 2 discriminative model. To use the nomogram, draw a vertical line from each variable’s value to the “Points” row to obtain individual scores. Sum these scores to get the “Total Points”. Then, draw a vertical line from the “Total Points” axis to the bottom axis to estimate the risk of PLOS. ROC curve, Receiver Operating Characteristic Curve; SOFA, Sequential Organ Failure Assessment; RBC, Red Blood Cell; MV, Mechanical Ventilation; AKI, Acute Kidney Injury; CRRT, Continuous Renal Replacement Therapy; PLOS, Prolonged Length Of Stay; HR, Heart Rate; SBP, Systolic Blood Pressure.

Variables with baseline differences were preliminarily screened using LASSO regression analysis, and a total of 10 variables were selected (including AKI, vasopressor, MV, CRRT, HR, SBP, potassium, RBC, SOFA, and age) (Fig. 2A‒B). Analysis of collinearity for the above 10 variables showed that there was no collinearity between the variables (All VIF < 10) (Table S4). These variables were included in multivariable logistic regression models (Table 1). In model 1, the results indicated that potassium, RBC, age, HR, and AKI might be independent influencing factors for PLOS (all p < 0.05). In model 2, SOFA, potassium, RBC, age, AKI, SBP, vasopressor, MV, and CRRT were associated with PLOS (all p < 0.05). Based on the different variables in model 1 and model 2, nomogram 1 (Fig. 3A) and nomogram 2 (Fig. 3B) were constructed. It was found that the discriminative performance of nomogram 1 (AUC = 0.797, Fig. 3C) for PLOS was lower than that of nomogram 2 (AUC = 0.854, Fig. 3D). Additionally, the calibration curve results for nomogram 2 indicated good model performance (Fig. 3E), with a Brier Score of 0.127.

**Table 1:** The results of the multivariable logistic regression analysis.

Variable	OR (95% CI)	p
Model 1		
(Intercept)	23.748 (4.979–115.118)	< 0.001
Age	0.986 (0.977–0.995)	0.002
SOFA	0.986 (0.936–1.038)	0.596
HR	1.007 (1.000–1.014)	0.038
Potassium	0.284 (0.208–0.384)	<0.001
RBC	0.419 (0.336–0.518)	<0.001
SBP	1.005 (0.998–1.013)	0.165
AKI (Yes)	9.091 (5.595–15.766)	<0.001
Model 2		
(Intercept)	3.266 (0.579–18.554)	0.181
Age	0.989 (0.979–0.999)	0.026
SOFA	0.888 (0.835–0.943)	<0.001
HR	1.005 (0.997–1.012)	0.228
Potassium	0.302 (0.215–0.420)	<0.001
RBC	0.524 (0.413–0.660)	<0.001
SBP	1.016 (1.008–1.025)	<0.001
AKI (Yes)	6.192 (3.737–10.917)	<0.001
CRRT (Yes)	3.780 (2.486–5.811)	<0.001
Vasopressor (Yes)	4.649 (3.435–6.317)	<0.001

OR, Odds Ratio; CI, Confidence Interval; SOFA, Sequential Organ Failure Assessment; RBC, Red Blood Cell; MV, Mechanical Ventilation; AKI, Acute Kidney Injury; CRRT, Continuous Renal Replacement Therapy; HR, Heart Rate; SBP, Systolic Blood Pressure.

### Sensitivity analysis

The study showed that the AUC value of the nomogram 2 model for distinguishing 14-day PLOS was 0.858 (Fig. S1A), and the Brier Score of the calibration curve (Fig. S1B) was 0.103. In dataset 2, the AUC value of the nomogram 2 model for distinguishing PLOS using the 75th LOS as the threshold was 0.851 (Fig. S1C), and the Brier Score of the calibration curve (Fig. S1D) was 0.114; the AUC value for distinguishing PLOS at 14 days was 0.853 (Fig. S1E), and the Brier Score of the calibration curve (Fig. S1F) was 0.087. In summary, it can be seen that the nomogram 2 model performs well in distinguishing PLOS, regardless of whether the cutoff was set at the 75th LOS or 14-day LOS.

### Optimal associative model identification

**Fig. 4:**
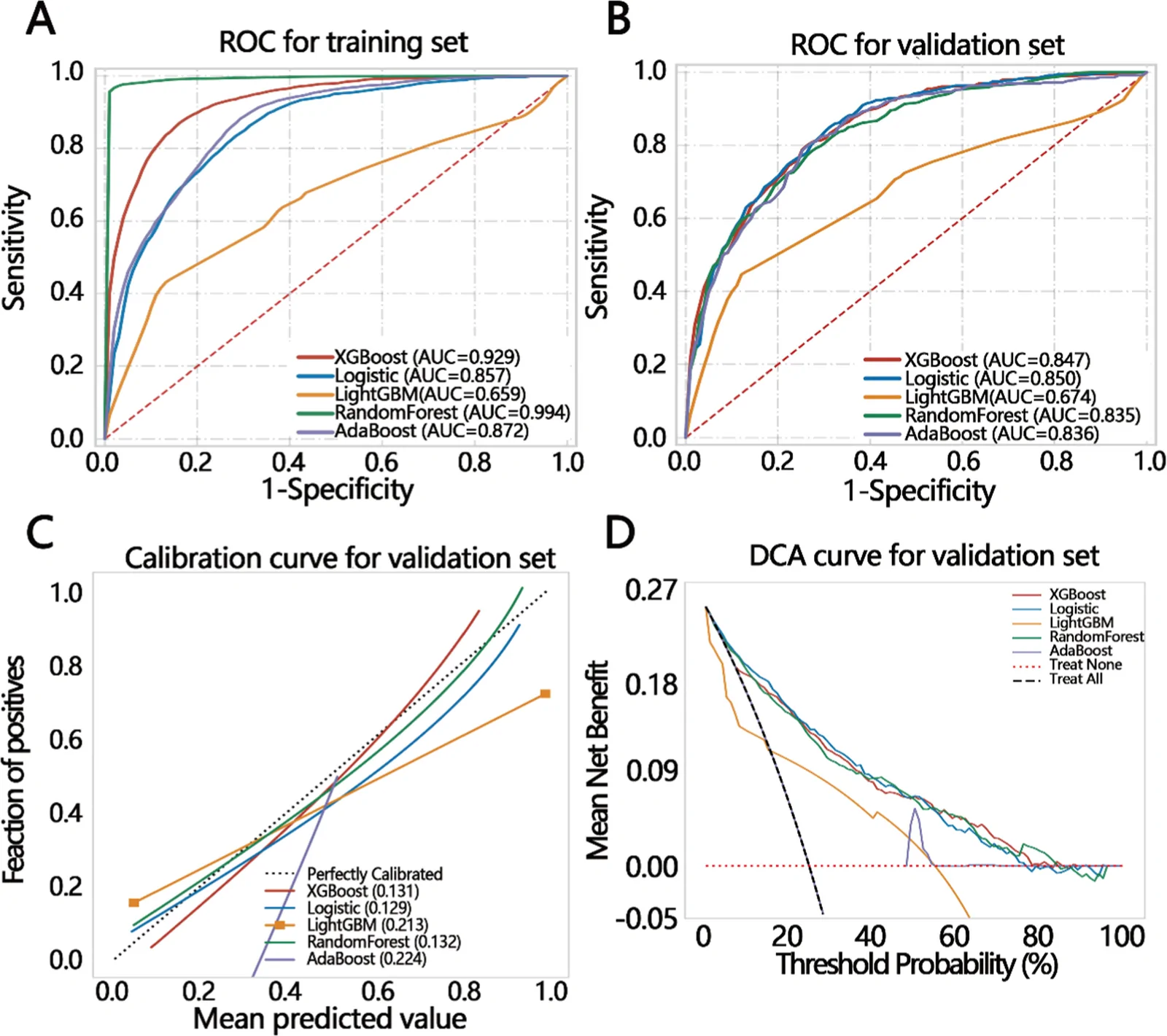
Identification of optimal associative models through 10-fold nested cross-validation. (A) Comparison of ROC curves for the training set. (B) Comparison of ROC curves in the validation set. (C) Comparison of multiple ML calibration curves in the validation set. (D) Comparison of DCA in the validation set. AUC, Area Under the Curve; ROC, Receiver Operating Characteristic; ML, Machine Learning; DCA, Curves and Decision Curve Analysis.

The AUCs of the baseline-only model (predictive) and the baseline and treatment model (associative) were 0.798 and 0.854 (Table S5). Therefore, the authors selected model 2, which included baseline data and treatment information, for further analysis. This study further evaluated whether the logistic model constructed based on 10 variables was optimal by comparing the logistic regression model with four other common ML models. Table 2 displays the results of 10-fold nested cross-validation for each model in terms of AUC, accuracy, sensitivity, specificity, PPV, NPV, and F1 score. Logistic regression exhibited the best generalization capability, with a training AUC of 0.857 and a validation AUC of 0.850, representing the smallest decrease (only 0.007) among all ML models. In terms of key validation set performance indicators, Logistic regression achieved the highest values for AUC (0.850), sensitivity (0.833), and NPV (0.927), demonstrating stable and balanced discriminative performance. Fig. 4A and 4B illustrate the ROC curves of the five models on the training and validation sets. Fig. 4C compares the calibration curve of each ML model in the validation set. The DCA results demonstrate that the clinical net benefit probability threshold for the logistic model was near 8%‒79% (Fig. 4D). Overall, the Logistic regression model performed robustly on multiple assessment dimensions and may outperform other compared ML models.

**Table 2:** Results of 10-fold nested cross-validation for five ML models.

Data set	AUC (95% CI)	Accuracy (95% CI)	Sensitivity (95% CI)	Specificity (95% CI)	PPV (95% CI)	NPV (95% CI)	F1 score (95% CI)
Training set							
XGBoost	0.929 (0.914–0.944)	0.853 (0.843–0.863)	0.871 (0.861–0.881)	0.847 (0.831–0.863)	0.653 (0.630–0.676)	0.953 (0.950–0.955)	0.746 (0.734–0.758)
Logistic	0.857 (0.834–0.879)	0.744 (0.734–0.754)	0.851 (0.829–0.872)	0.709 (0.689–0.730)	0.491 (0.480–0.502)	0.936 (0.929–0.943)	0.622 (0.618–0.625)
LightGBM	0.659 (0.626–0.692)	0.751 (0.707–0.796)	0.523 (0.451–0.595)	0.826 (0.750–0.903)	0.531 (0.473–0.589)	0.842 (0.830–0.854)	0.511 (0.473–0.549)
RandomForest	0.994 (0.989–0.998)	0.982 (0.981–0.983)	0.943 (0.939–0.947)	0.995 (0.994–0.996)	0.983 (0.980–0.986)	0.982 (0.980–0.983)	0.963 (0.960–0.965)
AdaBoost	0.872 (0.851–0.892)	0.760 (0.750–0.770)	0.868 (0.847–0.889)	0.725 (0.705–0.745)	0.510 (0.497–0.522)	0.944 (0.937–0.951)	0.642 (0.637–0.646)
Validation set							
XGBoost	0.847 (0.776–0.918)	0.776 (0.753–0.800)	0.714 (0.674–0.754)	0.797 (0.766–0.827)	0.540 (0.504–0.576)	0.895 (0.881–0.909)	0.613 (0.583–0.643)
Logistic	0.850 (0.780–0.919)	0.731 (0.700–0.761)	0.833 (0.808–0.858)	0.697 (0.655–0.739)	0.480 (0.448–0.511)	0.927 (0.917–0.938)	0.607 (0.580–0.633)
LightGBM	0.674 (0.572–0.776)	0.754 (0.701–0.806)	0.552 (0.454–0.650)	0.820 (0.728–0.912)	0.544 (0.475–0.613)	0.852 (0.833–0.871)	0.527 (0.476–0.577)
RandomForest	0.835 (0.761–0.910)	0.813 (0.796–0.829)	0.408 (0.357–0.460)	0.945 (0.935–0.956)	0.707 (0.656–0.758)	0.830 (0.817–0.843)	0.515 (0.463–0.568)
AdaBoost	0.836 (0.762–0.911)	0.733 (0.706–0.760)	0.808 (0.758–0.857)	0.708 (0.667–0.749)	0.481 (0.452–0.510)	0.920 (0.903–0.936)	0.600 (0.574–0.625)

ML, Machine Learning; AUC, Area Under the Curve; PPV, Positive Prediction Value; NPV, Negative Prediction Value.

### Optimal associative model validation based on the 10-fold cross-validation method

**Fig. 5:**
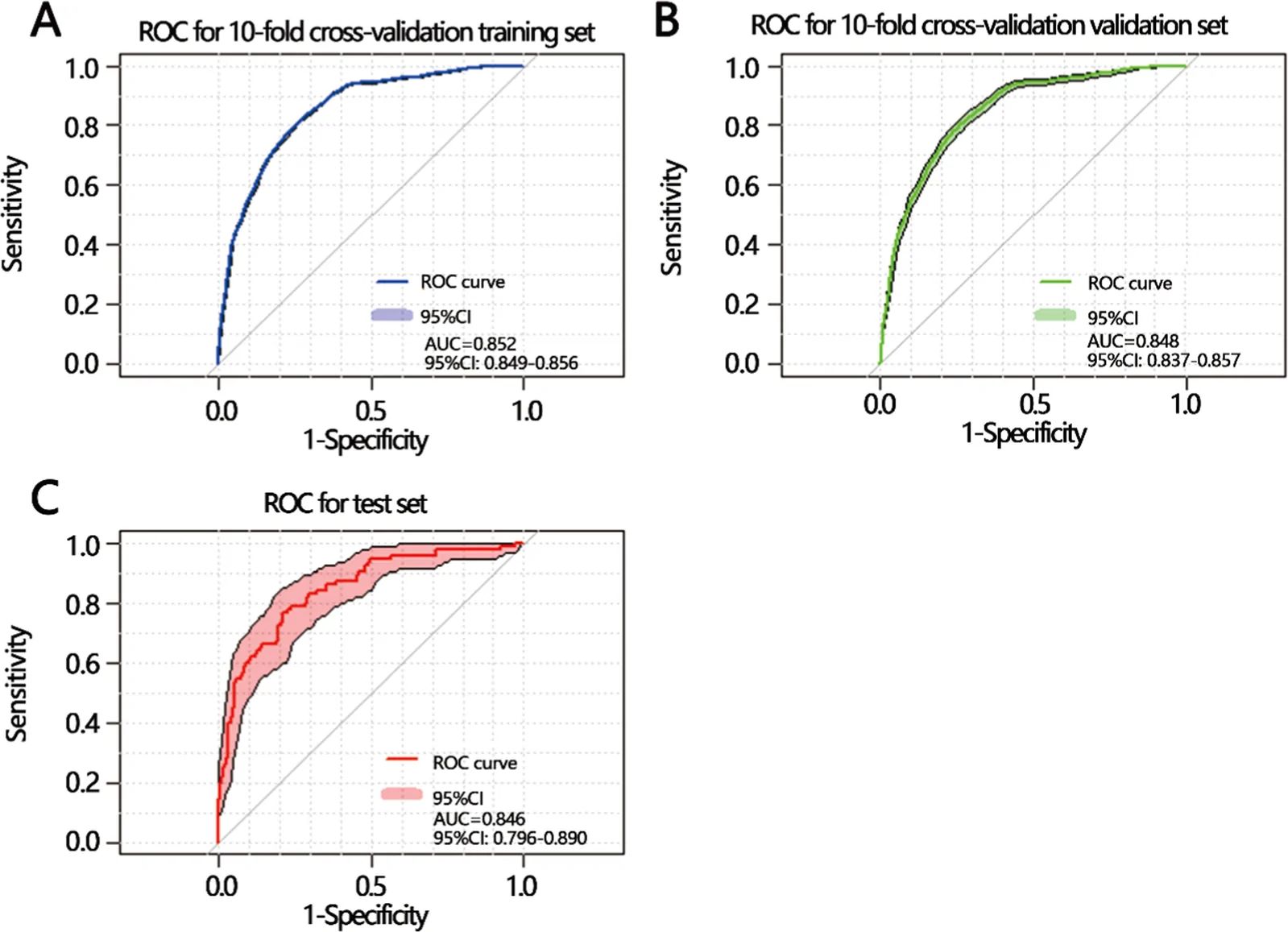
The ROC results of 10-fold cross-validation repeated 5-times for the associative model. (A) The mean AUC for the training sets. (B) The mean AUC for the validation sets. (C) The ROC curve of the test set. AUC, Area Under the Curve; ROC, Receiver Operating Characteristic.

The stability of discriminative performance for the optimal associative logistic regression model was validated using 10-fold cross-validation repeated 5-times (Table S6). Results indicated that the 10-fold cross-validation repeated 5-times yielded mean AUC values of 0.852 (0.849‒0.856) and 0.848 (0.837‒0.857) for the training and validation sets, respectively, with a test set AUC of 0.846 (0.796‒0.890) (Fig. 5A‒C).

### The interpretation of the predictive model

**Fig. 6:**
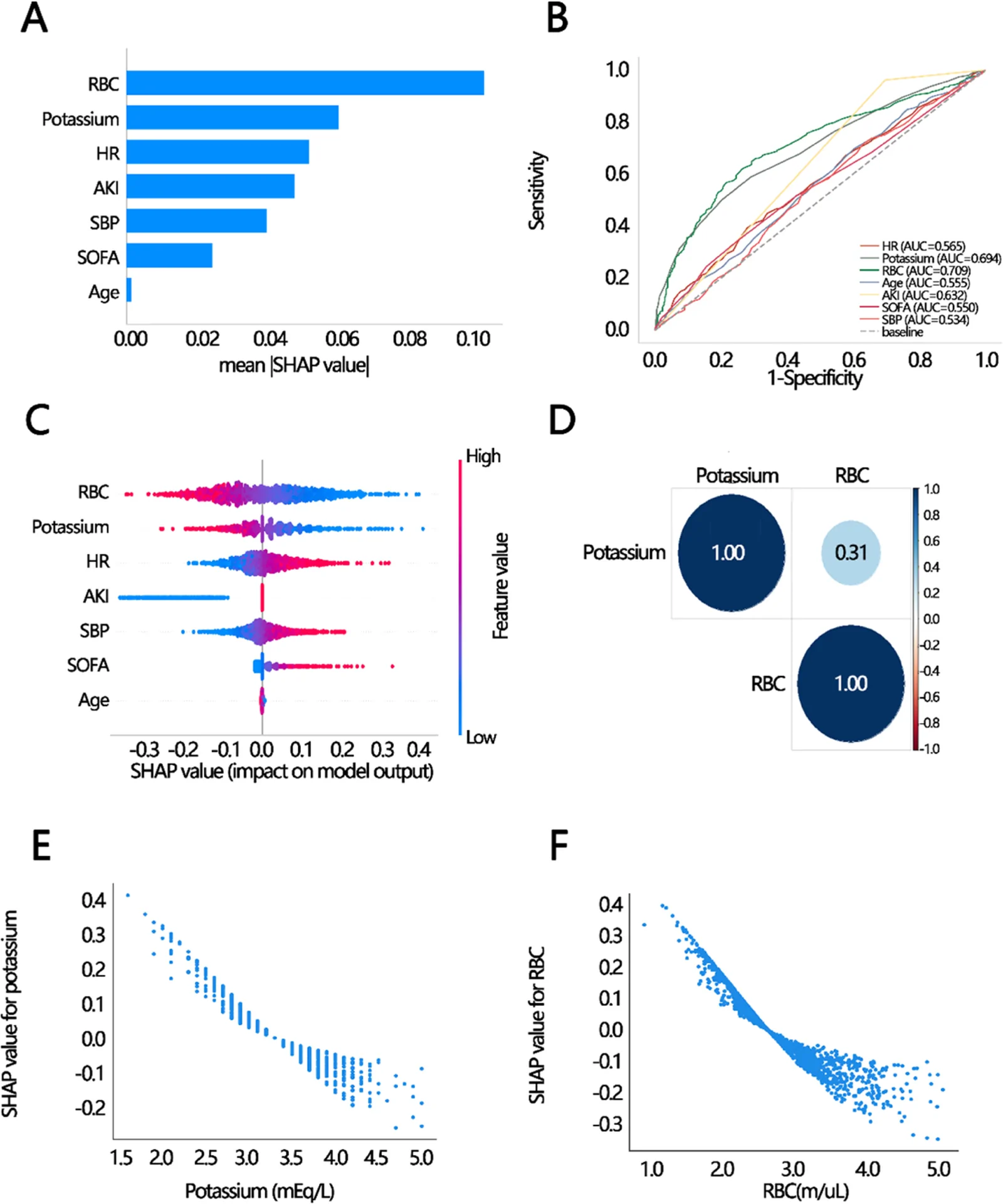
Interpretation of the SHAP model. (A) Bar graph. (B) Comparison of ROC for 7 variables. (C) The SHAP summary plot. (D) Spearman correlation heat map of potassium and RBC. (E) Dependence plot of potassium versus SHAP values. (F) Dependence plot of RBC versus SHAP values. In the dependence plots, negative SHAP values reveal a low likelihood of PLOS, while positive SHAP values reveal a high likelihood of PLOS. Note: The SHAP values were derived from the baseline-only model. SHAP, Shapley Additive explanations algorithm; SOFA, Sequential Organ Failure Assessment; RBC, Red Blood Cell; AKI, Acute Kidney Injury; PLOS, Prolonged Length Of Stay; HR, Heart Rate; SBP, Systolic Blood Pressure.

**Fig. 7:**
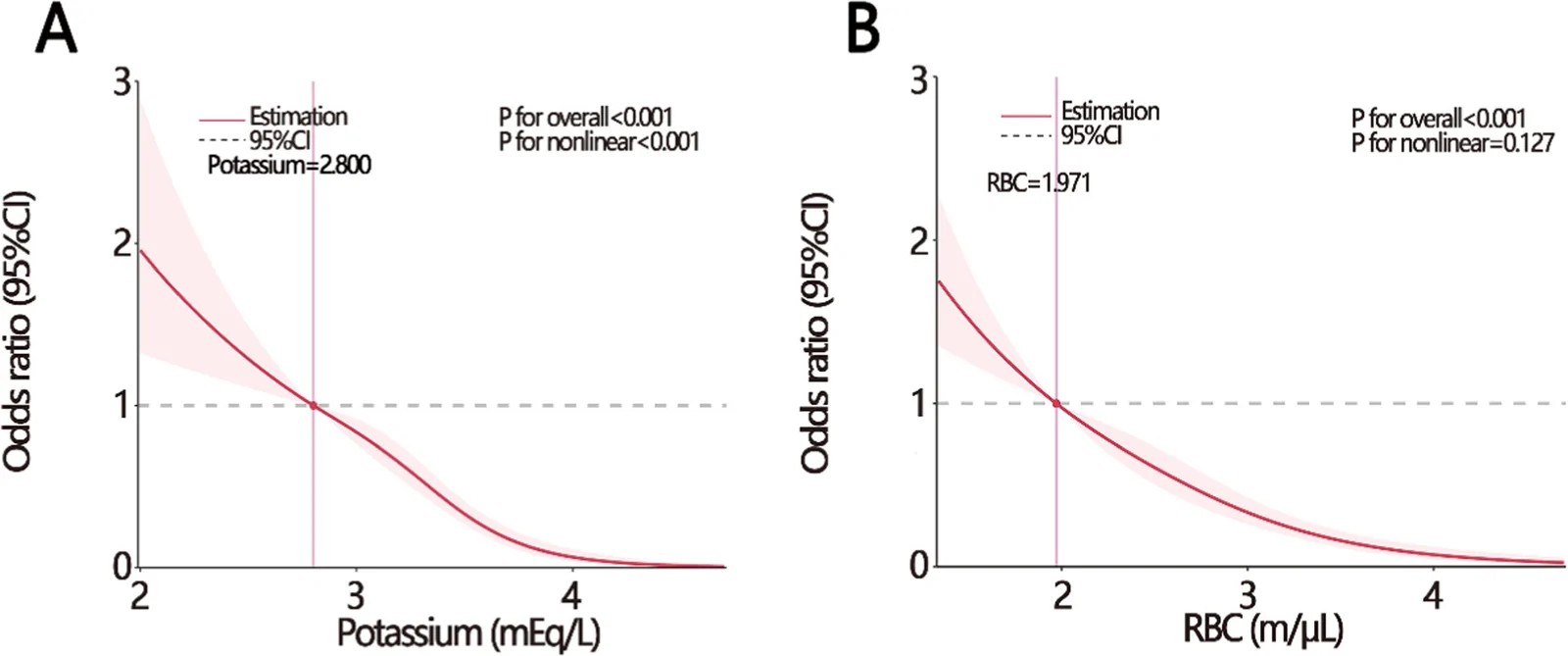
The non-linear relationship between potassium levels and RBC with PLOS. (A) The RCS curve between potassium levels and the risk of PLOS. (B) The RCS curve between RBC levels and the risk of PLOS. PLOS, Prolonged Length of Stay; RBC, Red Blood Cells; RCS, Restricted Cubic Spline Curve.

Subsequently, the logistic regression model, only containing baseline information, was interpreted using the SHAP method. The authors calculated the contribution of each feature to the predictive model output to identify the most relevant predictive factors. The higher the SHAP value of a feature, the greater its impact on the model. Fig. 6A displays the ranking of variable importance within the predictive model. Potassium and RBC exhibited high AUC values of 0.694 and 0.709, while AKI demonstrated a high sensitivity of 0.962 (Table S7 and Fig. 6B). The summary plot revealed a wide range of SHAP values for potassium with a monotonically decreasing trend (Fig. 6C). Moreover, the SHAP values of potassium and RBC showed a similar pattern in the swarm plot (Fig. 6C), which may suggest an interaction between the two variables. Further analyses were conducted to verify this interaction, but it was found to be nonexistent (Table S8). Spearman’s correlation results showed a weak positive correlation between potassium and RBC (*r* = 0.31, p < 0.05), suggesting that the two may have similar trends (Fig. 6D). The dependency graph and RCS analyses also confirmed this assumption. Dependency plot results indicated that SHAP values decreased as potassium or RBC levels increased (Fig. 6E‒F). Similarly, RCS results demonstrated a negative association between potassium levels and RBC with PLOS risk (all p for overall < 0.001) (Fig. 7A‒B). The authors transformed potassium and RBC levels into binary variables using the cut-off values of RCS analysis (2.800 mEq/L of potassium and 1.971 m/μL of RBC). Results indicated that both low potassium levels (< 2.800 mEq/L) (OR = 2.941, 95% CI 2.113‒4.093) and low RBC (< 1.971 m/μL) (OR = 2.077, 95% CI 1.507‒2.864) were risk factors for PLOS in sepsis patients combined with MI (Table S9). Sensitivity analysis validated the influence of low potassium levels on PLOS (Table S10).

## Discussion

This study aimed to identify associative factors of PLOS in sepsis patients combined with MI and construct an associative model based on ML algorithms. Through the analysis, the authors identified key clinical indicators that were closely associated with PLOS in sepsis patients combined with MI, including age, SOFA, potassium, RBC, AKI, vasopressor, MV, SBP, and CRRT. It was found that Logistic regression models outperformed other ML models in discriminating PLOS in sepsis patients combined with MI.

Although models for sepsis detection, risk assessment, and prognostic discrimination have been created, reliable discriminative models for PLOS in sepsis patients combined with MI remain scarce. Previous studies have shown significant potential for the application of ML in related areas. A retrospective cohort study involving 2103 sepsis patients was used to evaluate six ML algorithms to discriminate acute myocardial infarction, and the results demonstrated that the gradient boosting classifier performed best in terms of discriminative power, calibration, and clinical applicability.^21^ Another study on sepsis patients combined with MI found that the XGBoost model demonstrated superior performance in discriminating one-year mortality (test set AUROC = 0.83, 95% CI 0.79‒0.87).^22^ The optimal model varied across studies in discriminating between different clinical outcomes. Grovu R et al. utilized ML to discriminate LOS in patients with lupus flare-ups and found that the XGBoost model performed the best (AUC = 0.87).^23^ Conversely, another study involving elderly hip fracture patients showed that support vector machine and Logistic regression were more advantageous in discriminating PLOS.^24^ The results of the present study were consistent with the findings of these studies, as the logistic regression model showed high stability and discriminative accuracy in discriminating PLOS in sepsis patients combined with MI. The model has high sensitivity and can accurately identify high-risk patients. In this study, although prediction models based solely on baseline information (AUC = 0.798) may exhibit lower performance than the associative model incorporating baseline and treatment information (AUC = 0.854), their value in clinical practice remains unique and irreplaceable. The model’s core strengths lie in its time-specificity and decision-oriented nature. It enables clinicians to receive early warnings based purely on patients’ baseline risk at the earliest possible stage before medical interventions commence, thereby facilitating genuine early identification of high-risk patients and pre-allocation of resources. Notably, in the present cohort, the static SOFA alone demonstrated limited discriminative ability for PLOS (AUC = 0.550), whereas the baseline model achieved an AUC of 0.798, suggesting that combining the static SOFA with other admission variables may offer better risk stratification for this specific outcome. However, given the retrospective nature of this study, a direct comparison with dynamic SOFA assessments requires further validation in future prospective studies. This study further highlighted the potential application of ML in medical LOS discrimination and advocated for further research to enhance model performance and clinical applicability.

In this study, factors such as SBP, SOFA, potassium, RBC, age, AKI, vasopressor, MV, and CRRT were significantly linked to PLOS in sepsis patients combined with MI. SOFA score, as a key indicator for assessing the severity of sepsis and organ dysfunction, is an independent discriminative factor of LOS.^25^ Research has demonstrated that the duration of MV use in patients with septic cardiomyopathy was prolonged.^26^ Although MV improves oxygenation, it may also trigger ventilator-associated lung injury. When used with sedative medications, MV may increase the risk of delirium and prolong LOS in the ICU.^27^ AKI is a common complication of sepsis that significantly increases the risk of poor prognosis, with Renal Replacement Therapy (RRT) being the primary intervention for sepsis-related AKI.^28^ Sepsis patients combined with MI who also experienced AKI may have worse clinical outcomes, leading to longer LOS. The use of vasopressor drugs reflects the patients' hemodynamic instability and the need to maintain tissue perfusion.^29^ Their use is a side-effect of the severity of the disease and longer LOS in sepsis patients combined with MI. Furthermore, this study emphasized that blood potassium levels and RBC were crucial factors related to PLOS, and blood potassium levels were more important than SOFA. The core mechanisms of sepsis-induced MI include inflammation-mediated myocardial suppression, calcium regulation disorders, and mitochondrial dysfunction,^30^^,^^31^ while potassium imbalance further disrupts myocardial electrophysiological stability-hyperkalaemia inhibits conduction, and hypokalaemia induces arrhythmias, thereby exacerbating cardiac dysfunction,^32^^,^^33^ prolonging ICU LOS. In contrast, while the SOFA score reflects the severity of multi-organ failure, it lacks sensitivity to myocardial-specific damage (e.g., the cardiovascular SOFA is based solely on blood pressure and vasoactive drug usage),^34^ making it difficult to capture the unique effects of potassium imbalance on the myocardium. Therefore, its contribution to the model in this study is less than that of potassium. Lower potassium or RBC levels increased the likelihood of PLOS in sepsis patients combined with MI. One study showed that lower potassium concentrations were linked to longer LOS.^35^ There is a U-shaped relationship between serum potassium levels and mortality in the sepsis ICU population, with both hypokalemia and hyperkalemia associated with poor outcomes.^36^ It has been reported that compared to patients with normal potassium levels, those with serum potassium levels below 3.5 mmoL/L have a threefold higher risk of mortality. Additionally, hypokalemia is associated with primary aldosteronism and related hypertension, and primary aldosteronism has been shown to independently increase the risk of cardiac and renal injury.^37^ Mild low potassium may lead to weakness and myalgias, while severe low potassium can induce fatal arrhythmias.^38^ Similarly, studies have confirmed that anemia is significantly associated with PLOS.^39^ The reason for this may be that anemia leads to reduced mobility, impaired balance, and functional limitations in patients.^40^

These factors highlight the seriousness of the disease and the involvement of multiple organs in sepsis patients combined with MI. To enhance the clinical management of sepsis patients combined with MI and reduce their LOS, the following comprehensive interventions were advised. Hypokalaemia may exacerbate the risk of arrhythmia, requiring close monitoring of blood potassium levels and timely supplementation, as well as investigation of possible etiologies. Low RBC suggested anemia and should be assessed for nutritional deficiencies or inflammation inhibiting hematopoiesis, with transfusion of RBC or iron/ erythropoietin supplementation as necessary to improve myocardial oxygen supply. For patients identified as high-risk by the baseline model, closer monitoring and early multidisciplinary planning may help mitigate factors associated with PLOS.

This study successfully identified the key associative factors of PLOS in sepsis patients combined with MI through systematic analysis and verified the reliability and validity of Logistic regression models in the discrimination of PLOS in such patients. However, several limitations of this study required attention: 1) The MIMIC-IV database used in the study was derived from a single medical center in the United States only. Thus, population characteristics, treatment patterns, ICU admission criteria, and discharge practices may vary across different healthcare systems and regions, which limits the generalizability of the present findings. External validation using multi-center, international cohorts is therefore necessary before any clinical application can be considered. 2) This study employed a single cTnT value ≥0.01 ng/mL as the criterion, which may lead to an underestimation or misclassification. 3) As the study was designed as a retrospective analysis, the authors could only observe associations between variables without establishing precise causal relationships, necessitating prospective longitudinal studies to further validate these associations and their dynamic changes in the future. 4) Although data interpolation was used to address missing values, this method may not fully restore the true data distribution, potentially introducing residual bias in the model. 5) Since the time offset of MIMIC-IV was not publicly available, it is not possible to obtain the actual admission time of patients, and therefore, time validation cannot be performed. However, the authors internally validated the stability of the optimal model through 10-fold cross-validation repeated five times. 6) SHAP values revealed associations rather than causality. Furthermore, feature importance is model-specific and does not imply clinical primacy. It may be influenced by feature collinearity. Therefore, the authors must exercise caution when interpreting results, incorporating clinical knowledge. Despite this limitation, the remarkable stability and favorable calibration demonstrated by logistic regression establish it as a robust and interpretable tool, constituting an effective and valuable discovery in this clinical prediction scenario.

## Conclusion

In summary, SBP, SOFA, potassium, RBC, AKI, vasopressor, MV, age, and CRRT were associated with PLOS in sepsis patients combined with MI. The associative model the authors developed may aid in clinically identifying high-risk patients at risk of PLOS. However, its clinical utility requires further validation through prospective studies. Future interventional studies are needed to assess whether correcting these factors can genuinely improve patient outcomes, thereby providing a more reliable evidence base for clinical decision-making.

## Data availability

Only publicly available MIMIC-IV data were utilized in this study. This data can be found here: https://physionet.org/content/mimiciv/3.1/.

## Ethics approval

The MIMIC-IV database has been rigorously de-identified with all patient information before public release to ensure that patient privacy is fully protected. Therefore, there is no need to obtain ethical review board approval and informed patient consent.

## Authors’ contributions

Dabang Lei and Xianfei Ke contributed to the conception and design. Dabang Lei, Minglang Liao and Kai Yang contributed to the collection and assembly of data. Xianfei Ke, Minglang Liao and Kai Yang analyzed and interpreted the data. All authors wrote and approved the final manuscript.

## Declaration of competing interest

The authors declare no conflicts of interest.

## Appendix Supplementary materials

Image, application 1

## Footnotes

Supplementary material associated with this article can be found, in the online version, at doi:10.1016/j.clinsp.2026.101074.
