# Supplementary material for: Construction of an associative model for prolonged intensive care unit stay in sepsis patients combined with myocardial injury
Source: Clinics (Sao Paulo). 2026 Aug 13;81:101074. doi: 10.1016/j.clinsp.2026.101074 (PMC13495623; doi:10.1016/j.clinsp.2026.101074)
Supplement: Supplementary file 1 [file mmc1.docx]

**CLINICS-D-25-00450_Supplementary Material**

**Supplementary Table 1** Distribution of continuous variables in participant characteristics before imputation.

| **Variable** | **Missing (%)** | **Total (n=1792)** | **non-PLOS (n=1344)** | **PLOS (n=448)** | **p** |
| --- | --- | --- | --- | --- | --- |
| Age, years | 0 (0.000) | 59.000 [49.000, 68.000] | 60.000 [50.000, 68.000] | 57.000 [46.000, 67.000] | <0.001 |
| BMI, kg/m^2^ | 0 (0.000) | 27.200 [23.400, 32.600] | 27.200 [23.500, 32.800] | 27.400 [23.100, 32.400] | 0.578 |
| SOFA | 0 (0.000) | 3.000 [2.000, 5.000] | 3.000 [2.000, 4.000] | 4.000 [2.000, 5.000] | 0.001 |
| SAPS II | 0 (0.000) | 39.000 [30.000, 49.000] | 38.000 [29.000, 47.000] | 42.000 [33.000, 55.000] | <0.001 |
| HR, beats/min | 1 (0.056) | 75.805±17.380 | 74.779±17.145 | 78.890±17.713 | <0.001 |
| SBP, mmHg | 5 (0.279) | 86.000 [77.000, 97.000] | 86.000 [78.000, 97.000] | 84.000 [76.000, 97.000] | 0.029 |
| DBP, mmHg | 5 (0.279) | 46.000 [39.000, 53.000] | 46.000 [39.000, 53.000] | 46.000 [39.000, 53.000] | 0.769 |
| SpO_2_, % | 2 (0.112) | 92.000 [89.000, 95.000] | 92.000 [90.000, 95.000] | 92.000 [88.000, 95.000] | 0.205 |
| RR, breaths/min | 250 (13.951) | 13.000 [11.000, 16.000] | 13.000 [10.000, 15.000] | 13.000 [11.000, 16.000] | 0.182 |
| Glucose, mg/dL | 2 (0.112) | 105.000 [87.000, 126.000] | 105.000 [88.000, 125.000] | 104.000 [86.000, 127.000] | 0.611 |
| Potassium, mEq/L | 0 (0.000) | 3.300 [3.000, 3.600] | 3.400 [3.100, 3.600] | 3.100 [2.800, 3.300] | <0.001 |
| pH | 284 (15.848) | 7.310 [7.220, 7.380] | 7.320[ 7.230, 7.390] | 7.270 [7.180, 7.360] | <0.001 |
| PaO_2_, mmHg | 284 (15.848) | 57.000 [38.000, 87.000] | 57.000 [38.000, 88.000] | 57.000 [40.000, 84.000] | 0.880 |
| PCO_2_, mmHg | 284 (15.848) | 35.000 [30.000, 41.000] | 35.000 [30.000, 41.000] | 34.000 [29.000, 40.000] | 0.167 |
| Platelets, K/μL | 7 (0.391) | 169.000 [102.000, 240.000] | 170.000 [102.000, 241.000] | 167.000 [103.000, 235.000] | 0.738 |
| WBC, K/μL | 8 (0.446) | 9.400 [6.100, 13.400] | 9.100 [6.000, 13.000] | 10.500 [6.200, 14.500] | 0.013 |
| AG, mEq/L | 6 (0.335) | 13.000 [11.000, 16.000] | 13.000 [11.000, 15.000] | 14.000 [11.000, 16.000] | 0.023 |
| Chloride, mEq/L | 4 (0.223) | 101.000 [97.000, 105.000] | 101.000 [97.000, 105.000] | 101.000 [97.000, 105.000] | 0.376 |
| Creatinine, mg/dL | 4 (0.223) | 0.900 [0.700, 1.600] | 1.000 [0.700, 1.700] | 0.900 [0.700, 1.500] | 0.056 |
| INR | 123 (6.864) | 1.200 [1.100, 1.500] | 1.200 [1.100, 1.500] | 1.200 [1.100, 1.500] | 0.410 |
| PT, second | 123 (6.864) | 13.800 [12.300, 16.300] | 13.800 [12.300, 16.300] | 13.700 [12.400, 16.400] | 0.747 |
| RBC, m/μL | 0 (0.000) | 2.660 [2.250, 3.250] | 2.840 [2.360, 3.380] | 2.310 [2.090, 2.640] | <0.001 |
| BUN, mg/ dL | 77 (4.297) | 12.000 [10.000, 20.000] | 13.000 [10.000, 20.000] | 11.000 [10.000, 17.000] | <0.001 |

PLOS, Prolonged Length Of Stay; BMI, Body Mass Index; SAPS II, Simplified Acute Physiology Score II; SOFA, Sequential Organ Failure Assessment; pH, Pondus Hydrogenii; RBC, Red Blood Cell; HR, Heart Rate; SBP, Systolic Blood Pressure; DBP, Diastolic Blood Pressure; SpO_2_, Percutaneous Arterial Oxygen Saturation; PaO_2_, Partial pressure of arterial oxygen; PCO_2_, Partial pressure of Carbon dioxide; RR, Respiratory Rate; WBC, White Blood Cell; AG, Anion Gap; INR, International Normalized Ratio; PT, Prothrombin Time; BUN, Blood Urea Nitrogen

**Supplementary Table 2** Distribution of categorical variables in participant characteristics.

| **Variable** | **Total (n=1792)** | **non-PLOS (n=1344)** | **PLOS (n=448)** | **p** |
| --- | --- | --- | --- | --- |
| Gender, n (%) |  |  |  |  |
| Male | 997 (55.636) | 740 (55.060) | 257 (57.366) | 0.395 |
| Female | 795 (44.364) | 604 (44.940) | 191 (42.634) |  |
| Septic shock, n (%) |  |  |  |  |
| No | 1347 (75.167) | 1065 (79.241) | 282 (62.946) | <0.001 |
| Yes | 445 (24.833) | 279 (20.759) | 166 (37.054) |  |
| AKI, n (%) |  |  |  |  |
| No | 422 (23.549) | 405 (30.134) | 17 (3.795) | <0.001 |
| Yes | 1366 (76.228) | 935 (69.568) | 431 (96.205) |  |
| Missing | 4 (0.223) | 4 (0.298) | 0 (0.000) |  |
| COPD, n(%) |  |  |  |  |
| No | 1333 (74.386) | 989 (73.586) | 344 (76.786) | 0.179 |
| Yes | 459 (25.614) | 355 (26.414) | 104 (23.214) |  |
| Diabetes, n (%) |  |  |  |  |
| No | 1327 (74.051) | 985 (73.289) | 342 (76.339) | 0.202 |
| Yes | 465 (25.949) | 359 (26.711) | 106 (23.661) |  |
| Vasopressor, n (%) |  |  |  |  |
| No | 1002 (55.915) | 859 (63.914) | 143 (31.920) | <0.001 |
| Yes | 790 (44.085) | 485 (36.086) | 305 (68.080) |  |
| Aspirin use, n (%) |  |  |  |  |
| No | 1216 (67.857) | 918 (68.304) | 298 (66.518) | 0.483 |
| Yes | 576 (32.143) | 426 (31.696) | 150 (33.482) |  |
| MV, n (%) |  |  |  |  |
| No | 1489 (83.092) | 1219 (90.699) | 270 (60.268) | <0.001 |
| Yes | 303 (16.908) | 125 (9.301) | 178 (39.732) |  |
| CRRT, n (%) |  |  |  |  |
| No | 1619 (90.346) | 1282 (95.387) | 337 (75.223) | <0.001 |
| Yes | 173 (9.654) | 62 (4.613) | 111 (24.777) |  |

PLOS, Prolonged Length Of Stay; MV, Mechanical Ventilation; AKI, Acute Kidney Injury; COPD, Chronic Obstructive Pulmonary Disease; CRRT, Continuous Renal Replacement Therapy.

**Supplementary Table 3** Distribution of difference continuous variables in participant characteristics after imputation.

| **Variable** | **Total (n=1792)** | **non-PLOS (n=1344)** | **PLOS (n=448)** | **p** |
| --- | --- | --- | --- | --- |
| HR, beats/min | 75.801±17.376 | 74.779±17.145 | 78.865±17.701 | <0.001 |
| SBP, mmHg | 86.000 [77.000, 97.000] | 86.000 [78.000, 97.000] | 84.000 [76.000, 97.000] | 0.034 |
| Potassium, mEq/L | 3.300 [3.000, 3.600] | 3.400 [3.100, 3.600] | 3.100 [2.800, 3.300] | <0.001 |
| pH | 7.313 [7.230, 7.374] | 7.320 [7.247, 7.380] | 7.280 [7.190, 7.360] | <0.001 |
| WBC, K/μL | 9.300 [6.100, 13.400] | 9.100 [6.000, 13.000] | 10.500 [6.400, 14.300] | 0.013 |
| AG, mEq/L | 13.000 [11.000, 15.000] | 13.000 11.000, 15.000] | 13.000 [11.000, 16.000] | 0.027 |
| RBC, m/μL | 2.660 [2.250, 3.250] | 2.840[2.360,3.380] | 2.310 [2.090, 2.640] | <0.001 |
| BUN, mg/ dL | 12.000 [10.000, 20.400] | 13.000 [10.000, 21.000] | 11.000 [10.000, 19.000] | <0.001 |
| SOFA | 3.000 [2.000, 5.000] | 3.000 [2.000, 4.000] | 4.000 [2.000, 5.000] | 0.001 |
| SAPS II | 39.000 [30.000, 49.000] | 38.000 [29.000, 47.000] | 42.000 [33.000, 55.000] | <0.001 |
| Age, years | 59.000 [49.000, 68.000] | 60.000 [50.000, 68.000] | 57.000 [46.000, 67.000] | <0.001 |

PLOS, Prolonged Length of Stay; SAPS II, Simplified Acute Physiology Score II; SOFA, Sequential Organ Failure Assessment; pH, Pondus Hydrogenii; RBC, Red Blood Cell; HR, Heart Rate; SBP, Systolic Blood Pressure; WBC, White Blood Cell; AG, Anion Gap; BUN, Blood Urea Nitrogen.

**Supplementary Table 4** The collinearity results of 10 preliminary screening variables.

| **Variable** | **VIF** |
| --- | --- |
| Vasopressor | 1.356 |
| SOFA | 1.283 |
| RBC | 1.272 |
| CRRT | 1.217 |
| SBP | 1.151 |
| Potassium | 1.135 |
| AKI | 1.121 |
| MV | 1.073 |
| Age | 1.062 |
| HR | 1.051 |

VIF, Variance Inflation Factor; SOFA, Sequential Organ Failure Assessment; RBC, Red Blood Cell; MV, Mechanical Ventilation; AKI, Acute Kidney Injury; CRRT, Continuous Renal Replacement therapy; HR, Heart Rate.

**Supplementary Table 5** The performance of the predictive model and associative model.

| **Model** | **AUC (95% CI)** |
| --- | --- |
| The baseline-only model (predictive) | 0.798 (0.777‒0.820) |
| The baseline and treatment model (associative) | 0.854 (0.834‒0.874) |

Note: The predictive model included potassium, RBC, age, SBP, HR, SOFA, and AKI. The associative model included the SOFA score, potassium, age, SBP, HR, RBC, AKI, vasopressor, MV, and CRRT.

AUC, Area Under the Curve; CI, Confidence Interval; RBC, Red Blood Cell; SOFA, Sequential Organ Failure Assessment; AKI, Acute Kidney Injury; MV, Mechanical Ventilation; CRRT, Continuous Renal Replacement Therapy; HR, Heart Rate; SBP, Systolic Blood Pressure.

**Supplementary Table 6** Results of 10-fold cross-validation for each repetition.

| **Repeated** | **AUC (95% CI) for training sets** | **AUC (95% CI) for validation sets** |
| --- | --- | --- |
| 1 | 0.8571 (0.8501‒0.8639) | 0.8522 (0.8287‒0.8723) |
| 2 | 0.8572 (0.8502‒0.8641) | 0.8497 (0.8281‒0.8712) |
| 3 | 0.8571 (0.8502‒0.8645) | 0.8513 (0.8302‒0.8717) |
| 4 | 0.8572 (0.8499‒0.8643) | 0.8499 (0.8274‒0.8690) |
| 5 | 0.8571 (0.8500‒0.8640) | 0.8508 (0.8290‒0.8732) |

AUC, Area Under the Curve; CI, Confidence Interval.

**Supplementary Table 7** The predictive performance of 7 variables on PLOS.

| **Variable** | **HR** | **Potassium** | **RBC** | **Age** | **AKI** | **SOFA** | **SBP** |
| --- | --- | --- | --- | --- | --- | --- | --- |
| AUC (95% CI) | 0.565 (0.536‒0.596) | 0.694 (0.667‒0.722) | 0.709 (0.686‒0.740) | 0.555 (0.532‒0.582) | 0.632 (0.618‒0.650) | 0.550 (0.524‒0.583) | 0.534 (0.504‒0.555) |
| Sensitivity (95% CI) | 0.393 (0.301‒0.725) | 0.589 (0.473‒0.607) | 0.657 (0.540‒0.681) | 0.697 (0.246‒0.865) | 0.962 (0.948‒0.981) | 0.344 (0.225‒0.556) | 0.712 (0.498‒0.806) |
| Specificity (95% CI) | 0.721 (0.398‒0.804) | 0.710 (0.688‒0.834) | 0.699 (0.658‒0.818) | 0.397 (0.233‒0.841) | 0.302 (0.281‒0.330) | 0.750 (0.562‒0.855) | 0.373 (0.255‒0.603) |
| Youden Index (95% CI) | 0.114 (0.084‒0.167) | 0.299 (0.262‒0.345) | 0.356 (0.322‒0.415) | 0.094 (0.063‒0.148) | 0.264 (0.237‒0.300) | 0.094 (0.064‒0.150) | 0.085 (0.051‒0.124) |
| Cutoff (95% CI) | 85.000 (70.000‒90.050) | 3.300 (3.300‒3.400) | 2.540 (2.500‒2.785) | 53.000 (45.000‒69.575) | 1.000 (1.000‒1.000) | 5.000 (4.000‒6.000) | 80.000 (75.325‒86.000) |
| Accuracy (95% CI) | 0.606 (0.477‒0.674) | 0.598 (0.552‒0.636) | 0.656 (0.604‒0.687) | 0.612 (0.402‒0.710) | 0.471 (0.447‒0.496) | 0.629 (0.552‒0.705) | 0.597 (0.516‒0.672) |
| Delong test | <0.001 | / | 0.376 | <0.001 | <0.001 | <0.001 | <0.001 |

PLOS, Prolonged Length Of Stay; AUC, Area Under the Curve; SOFA, Sequential Organ Failure Assessment; RBC, Red Blood Cell; AKI, Acute Kidney Injury; HR, Heart Rate; SBP, Systolic Blood Pressure.

**Supplementary Table 8** Interaction analysis results between potassium and RBC for PLOS.

| **Variable** | **OR (95% CI)** | **p** |
| --- | --- | --- |
| RBC | 0.757 (0.160‒3.561) | 0.725 |
| Potassium | 0.460 (0.131‒1.582) | 0.221 |
| RBC: Potassium | 0.802 (0.495‒1.295) | 0.368 |

PLOS, Prolonged Length Of Stay; OR, Odds Ratio; CI, Confidence Interval; RBC, Red Blood Cells.

**Supplementary Table 9** The impact of hypokalaemia and low RBC on PLOS.

| **Variable** | **OR (95% CI)** | **p** |
| --- | --- | --- |
| Potassium |  |  |
| ≥2.8 mEq/L | *Reference* |  |
| <2.8 mEq/L | 2.941 (2.113‒4.093) | <0.001 |
| RBC |  |  |
| ≥1.971 m/μL | *Reference* |  |
| <1.971 m/μL | 2.077 (1.507‒2.864) | <0.001 |

Note: Only one patient had a potassium level (5.7 mEq/L) above the normal range (3.5‒5.0 mEq/L).

RBC, Red Blood Cells; PLOS, Prolonged Length of Stay; OR, Odds Ratio; CI, Confidence Interval.

**Supplementary Table 10** Sensitivity analysis of potassium levels.

| **Variable** | **Adjusted OR (95% CI)** | **p** |
| --- | --- | --- |
| Potassium |  |  |
| ≥ 2.8 mEqL | *Reference* |  |
| < 2.8 mEqL | 2.234 (1.571‒3.178) | <0.001 |
| Potassium |  |  |
| ≥ 3.5 mEqL | *Reference* |  |
| < 3.5 mEqL | 3.570 (2.636‒4.834) | <0.001 |

Note: Only one patient had a potassium level (5.7 mEq/L) above the normal range (3.5‒5.0 mEq/L); AKI, diuretics and estimated glomerular filtration rate were adjusted.

RBC, Red Blood Cells; PLOS, Prolonged Length Of Stay; OR, Odds Ratio; CI, Confidence Interval; AKI, Acute Kidney Injury.

**Supplementary Figure**

**Figure S1** Sensitivity analysis. (A) ROC curve between nomogram 2 and PLOS with a 14-day LOS cutoff. (B) Calibration curve between nomogram 2 and PLOS with a 14-day LOS cutoff. (C) ROC curve between nomogram 2 and PLOS with a 75^th^ LOS cutoff in dataset 2. (D) Calibration curve between nomogram 2 and PLOS with a 75^th^ LOS cutoff in dataset 2. (E) ROC curve between nomogram 2 and PLOS with a 14-day LOS cutoff in dataset 2. (F) Calibration curve between nomogram 2 and PLOS with a 14-day LOS cutoff in dataset 2. ROC curve, Receiver Operating Characteristic curve; PLOS, Prolonged Length of Stay.
